# Sucrose Counteracts the Anti-Inflammatory Effect of Fish Oil in Adipose Tissue and Increases Obesity Development in Mice

**DOI:** 10.1371/journal.pone.0021647

**Published:** 2011-06-28

**Authors:** Tao Ma, Bjørn Liaset, Qin Hao, Rasmus Koefoed Petersen, Even Fjære, Ha Thi Ngo, Haldis Haukås Lillefosse, Stine Ringholm, Si Brask Sonne, Jonas Thue Treebak, Henriette Pilegaard, Livar Frøyland, Karsten Kristiansen, Lise Madsen

**Affiliations:** 1 Department of Biology, University of Copenhagen, Copenhagen, Denmark; 2 National Institute of Nutrition and Seafood Research (NIFES), Bergen, Norway; 3 Department of Exercise and Sport Sciences, University of Copenhagen, Copenhagen, Denmark; University of Hong Kong, China

## Abstract

**Background:**

Polyunsaturated n-3 fatty acids (n-3 PUFAs) are reported to protect against high fat diet-induced obesity and inflammation in adipose tissue. Here we aimed to investigate if the amount of sucrose in the background diet influences the ability of n-3 PUFAs to protect against diet-induced obesity, adipose tissue inflammation and glucose intolerance.

**Methodology/Principal Findings:**

We fed C57BL/6J mice a protein- (casein) or sucrose-based high fat diet supplemented with fish oil or corn oil for 9 weeks. Irrespective of the fatty acid source, mice fed diets rich in sucrose became obese whereas mice fed high protein diets remained lean. Inclusion of sucrose in the diet also counteracted the well-known anti-inflammatory effect of fish oil in adipose tissue, but did not impair the ability of fish oil to prevent accumulation of fat in the liver. Calculation of HOMA-IR indicated that mice fed high levels of proteins remained insulin sensitive, whereas insulin sensitivity was reduced in the obese mice fed sucrose irrespectively of the fat source. We show that a high fat diet decreased glucose tolerance in the mice independently of both obesity and dietary levels of n-3 PUFAs and sucrose. Of note, increasing the protein∶sucrose ratio in high fat diets decreased energy efficiency irrespective of fat source. This was accompanied by increased expression of *Ppargc1a* (peroxisome proliferator-activated receptor, gamma, coactivator 1 alpha) and increased gluconeogenesis in the fed state.

**Conclusions/Significance:**

The background diet influence the ability of n-3 PUFAs to protect against development of obesity, glucose intolerance and adipose tissue inflammation. High levels of dietary sucrose counteract the anti-inflammatory effect of fish oil in adipose tissue and increases obesity development in mice.

## Introduction

Today it is recognized that the potentially harmful effects of high fat diets relates to not only the amount, but also the type of dietary fatty acids. Whereas a high intake of saturated and trans fatty acids has been shown to be associated with increased risk of cardiovascular diseases in several studies, intake of polyunsaturated fatty acids (PUFAs) has been associated with lower cardiovascular risk [1], [2]. Thus, increasing the relative amount of PUFAs, both vegetable n-6 PUFAs and marine n-3 PUFA, at the expense of saturated fat is recommended. It is important to note, however, that more than 85% of the total dietary PUFA intake in Western diets today is vegetable n-6 PUFAs, mainly linoleic acid [3]. This is largely due to the high amount of linoleic acid in corn-, sunflower-, and soybean-oil used in both home-cooking and in industrially prepared food [4]. Moreover, animal feeds are enriched with n-6 PUFAs, and although meat production methods are diverse, meat fatty acid profiles will always reflect that of the animal feed [4]. Thus, the dietary n-3∶n-6 PUFA ratio has decreased [3], [4]. Although the exchange of saturated fat with vegetable n-6 PUFAs may have some beneficial effects on human health, a low n-3∶n-6 PUFAs ratio is associated with a high risk of several lipid-related disorders [2], [3]. A high intake of n-6 PUFAs has also been associated with childhood obesity, [4], [5]. Animal studies have shown that feeding mice a diet containing the n-6 PUFA, linoleic acid, during the pregnancy-lactation period leads to obesity in the offspring [6]. This effect, however, is prevented by inclusion of the n-3 PUFA α-linolenic acid in the diet [6]. These findings are in line with several studies demonstrating that dietary n-3 PUFAs are able to limit the development of diet-induced obesity [7]–[13].

Obesity may be considered as a state of chronic low-grade inflammation [14], [15]. Accumulated evidence strongly suggests that low grade chronic inflammation plays a crucial role in development of obesity related insulin resistance [16]. Furthermore, it should be noted that continuous subcutaneous infusion of lipopolysaccharide (LPS) is sufficient to induce adipose tissue inflammation, insulin resistance and obesity in mice [17]. It is also well documented that n-3 PUFAs are able to limit high fat diet-induced inflammation in adipose tissue in rodents [18]–[20]. Both n-3 PUFAs and n-6 PUFAs are substrats for cyclo- and lipoxygenases and n-3 PUFAs are traditionally assumed to act anti-innflammatory by competitive inhibition of the biosynthesis of arachidonic acid-derived pro-inflammatory prostaglandins of the 2-series and furthermore, n-3 PUFA-derived prostaglandins of the 3-series are believed to be less inflammatory [21], [22]. Recent research furthermore demonstrate that n-3 PUFAs may be converted to anti-inflammatory cyclooxygenase-2 derived electrophilic oxoderivatives and resolvins [21], [22]. Moreover, by activation of the fatty acid receptor, GPR120, n-3 PUFAs repress LPS- and TNFα-mediated inflammatory signalling responses, and thereby increase insulin sensitivity by repressing macrophage-induced adipose tissue inflammation [23]. Thus, consumption of n-6 PUFAs at the expense of n-3 PUFAs may aggravate the metabolic consequences of obesity. Increasing the dietary intake of n-3 PUFAs is therefore currently recommended by several health authorities.

In order to curb the increasing obesity problem, nutritionists and authorities have largely focused on reducing fat intake, as dietary fat contains more energy per gram than proteins and carbohydrates. As an alternative to low energy diets, low carbohydrate diets are becoming increasingly popular although still controversial. The mechanisms by which such diets induce weigh loss are still not fully elucidated, but it has been documented that high protein diets increase energy expenditure in part due to a thermic effect [24]. We have previously shown that the protein∶sucrose ratio in the background diet determines the adipogenic potential of dietary n-6 PUFAs in mice [25]. Mice fed n-6 PUFAs in combination with sucrose became obese, and had a markedly higher feed efficiency than mice *pair-fed* n-6 PUFAs in combination with proteins [25]. In fact, the high-protein fed mice needed almost 7 times more energy to achieve a weight gain of 1 g than mice on the high-sucrose diet [25]. The high protein diet led to an increased glucagon/insulin ratio, concomitant with elevated cAMP-dependent signaling, induction of COX-mediated prostaglandin synthesis and increased expression of uncoupling protein-1 (UCP1) in inguinal subcutaneous white fat [25]. In the present paper we aimed to investigate whether this phenomenon is restricted to n-6 PUFAs or if the effects of dietary fats, such as fish oils, which are considered beneficial to human health, also depend on the background diets. Furthermore, we aimed to examine whether the background diet exerts an influence on the ability of n-3 PUFAs to protect against glucose intolerance and adipose tissue inflammation.

## Results

### Sucrose counteracts the obesity-reducing effect of fish oil in *ad libitum* fed mice

**Figure 1:**
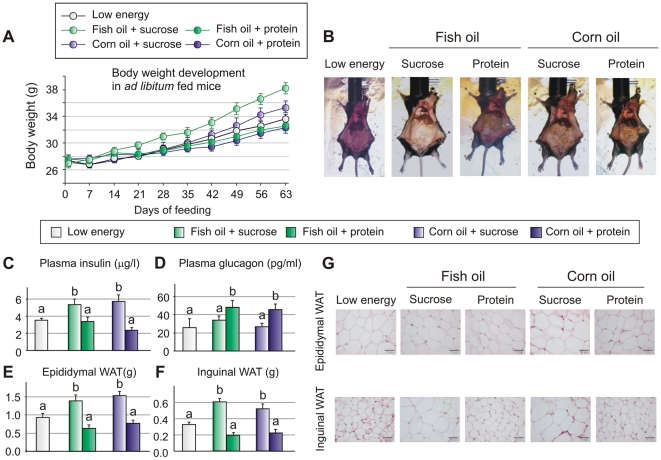
Sucrose counteracts the obesity-reducing effect of fish oil in *ad libitum* fed mice. Male C57BL/6 mice (n = 8) were fed isocaloric high fish oil or high corn oil diets with different carbohydrate and protein contents *ad libitum* for 9 weeks. A: Body weight development of *ad libitium* fed mice. (B) Prior to termination the mice were photographed. C–D: Insulin and glucagon levels were measured in plasma in the fed state. E–G: The weights of epididymal and inguinal white adipose tissues were recorded and sections were stained with hematoxylin and eosin. Data are presented as means ± SEM. Different small letters denote significant differences between the groups (P<0.05).

It is a general notion that intake of fish oil rich in n-3 PUFAs limits high fat diet-induced obesity in rodents, whereas diets rich in n-6 PUFAs have been associated with an increased propensity to develop obesity [6], [26]. As we have demonstrated that the obesogenic effect of n-6 PUFAs is determined by the content of carbohydrates and protein in the feed [25], we speculated whether the effect of dietary fats considered health-beneficial, such as fish oil, might be modulated by different background diets. To answer this question we fed C57BL/6J male mice isocaloric high fat diets (Table 1 and 2) containing corn oil or fish oil supplemented with either protein or sucrose or a conventional low fat diet *ad libitum* for 9 weeks. Contrasting the general notion that fish oil attenuates high fat diet-induced obesity, the mice fed the fish oil in combination with sucrose gained as much body weight as the mice fed corn oil and sucrose (Fig. 1A and B). When combined with sucrose, fish oil did not reduce the weights of neither epididymal (eWAT) nor inguinal white adipose tissue (iWAT) mass compared with corn oil (Fig. 1E and F). Moreover, morphological analyses demonstrated that the adipocyte size was similar in the two sucrose fed groups (Fig. 1G). Of note, weight gain in mice fed corn oil or fish oil plus protein were indistinguishable from that of mice fed the low fat diet (Fig. 1A). Compared with low fat fed mice, the weights and adipocytes sizes of eWAT and iWAT in mice fed both high fat diets in combination with protein tended to be smaller, but the differences did not reach statistical significance (Fig. 1E, F and G). Thus, when combined with a high intake of sucrose fish oil did not prevent obesity. However, high dietary protein content prevented weight gain and obesity when combined with either corn or fish oil.

**Table 1:** Macronutrient composition in the diets.

		High fish oil		High corn oil	
	Low energy	Sucrose	Protein	Sucrose	Protein
**Protein (g/kg)**	**200**	**200**	**540**	**200**	**540**
Casein	200	200	540	200	540
L-Cysteine	3	3	3	3	3
**Carbohydrate (g/kg)**	**619.5**	**439.5**	**99.5**	**439.5**	**99.5**
Corn starch	529.5	9.5	9.5	9.5	9.5
Sucrose	90	430	90	430	90
**Fat (g/kg)**	**70**	**250**	**250**	**250**	**250**
Soybean oil	70	70	70	70	70
Corn oil	-	-	-	180	180
Fish oil	-	180	180	-	-

**Table 2:** Fatty acid composition in the diets.

		High fish oil		High corn oil	
	Low energy	Sucrose	Protein	Sucrose	Protein
**SFA (mg/g)**	**9.6**	**53.3**	**50.3**	**31.4**	**31.4**
**MUFA (mg/g)**	**17.0**	**54.3**	**54.0**	**65.9**	**65.9**
**PUFA (mg/g)**	**36.2**	**100.6**	**99.5**	**120.8**	**121.2**
n-6 (mg/g)	32.7	40.6	38.9	117.3	117.8
n-3 (mg/g)	3.5	60.0	60.6	3.5	3.4
n-3/n-6	0.11	1.48	1.56	0.03	0.03

Abbreviations: SFA, saturated fatty acids; MUFA, monounsaturated fatty acids; PUFA, polyunsaturated fatty acids.

Sucrose, but not protein or fat, strongly stimulates pancreatic insulin secretion, and accordingly, plasma levels of insulin were consistently higher in mice fed the sucrose-based diets than in mice fed the protein-based diets (Fig. 1C). Conversely, the levels of plasma glucagon were lower and hence, the insulin∶glucagon ratio was about three times higher in mice fed high sucrose than in mice fed high protein irrespective of whether the diets contained corn oil or fish oil (Fig. 1D). Collectively, these results indicate that intake of sucrose and hence increased insulin secretion, abrogates the protective effects of fish oil in relation to adipocyte hyperplasia and hypertrophy and thereby the development obesity.

### Sucrose counteracts the anti-inflammatory effect of fish oil in adipose tissue

**Figure 2:**
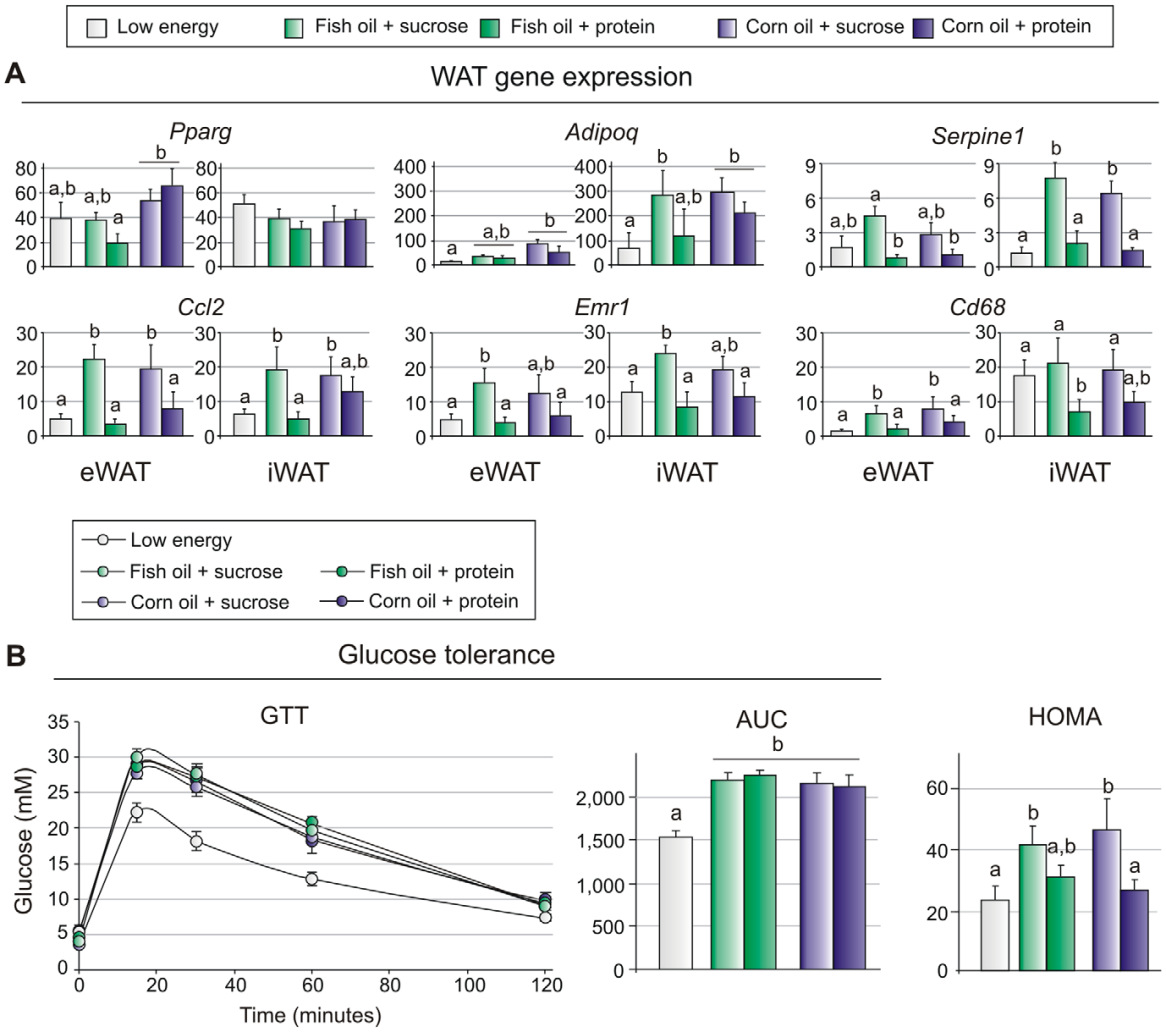
A high fat diet impairs glucose tolerance independent of macronutrient composition and obesity. A: Expressions of adipogenic and inflammatory marker genes (*Pparg* (peroxisome proliferator activated receptor γ), *Adipoq* (adiponectin), *Serpine1* (Plasminogen activator inhibitor-1), *Ccl2* (chemokine (C-C motif) ligand 2), *Emr1* (EGF-like module containing, mucin-like, hormone receptor-like sequence 1 or F4/80) and *Cd68* (CD68 antigen)) were measured in epididymal and inguinal white adipose tissue using RT-qPCR (n = 8). B: Intraperitoneal glucose tolerance test was performed in a separate set of mice (n = 10). Fasting glucose and insulin levels were measured to calculate HOMA-IR. Data are presented as means ± SEM. Different small letters denote significant differences between the groups, in 2A within the same tissue (P<0.05).

The ability of n-3 PUFAs to limit high fat diet-induced inflammation in adipose tissue is well documented [18]–[20]. As chronic low grade inflammation in adipose tissue is a characteristic trait of obesity [14], [15] and sucrose abrogates the anti-adipogenic effect of fish oil, we asked whether the background diet also attenuated the ability of n-3 PUFAs to protect against adipose tissue inflammation. Gene expression analyses of eWAT and iWAT revealed a striking correlation between macrophage- and inflammatory markers and the intake of sucrose-based diets irrespectively of the fat source (Fig. 2A). Expressions of macrophage marker genes *Emr1* (EGF-like module containing, mucin-like, hormone receptor-like sequence 1 or F4/80) and *Cd68*, as well as markers of inflammation *Serpine1* (Plasminogen activator inhibitor-1) and *Ccl2* (chemokine (C-C motif) ligand 2), were significantly higher in adipose tissue from mice fed sucrose than in mice fed high protein or a low fat diets (Fig. 2A). Moreover, we noticed a significant increase in the expression of *Pparg* (peroxisome proliferator-activated receptor γ) in eWAT in the mice fed protein supplemented with corn oil compared with mice fed protein supplemented with fish oil (Fig. 2A).

### A high fat diet impairs glucose tolerance independent of macronutrient composition and obesity

As adipose tissue inflammation is causally linked to development of insulin resistance and glucose intolerance, we subjected mice fed the different diets for 9 weeks to an intraperitoneal glucose tolerance test (GTT). Surprisingly, the GTT demonstrated that the glucose tolerance was impaired both in the mice fed the protein-based diets and in the mice fed the sucrose-based diets (Fig. 2B). Evidently, impaired glucose tolerance was dissociated from the state of obesity, suggesting that intake of relatively high amounts of fat reduces glucose tolerance even if weight gain and expression of inflammatory markers were maintained at low levels. However, fasting glucose and insulin levels were lower in mice fed high protein than high sucrose. Thus, calculation of HOMA-IR indicated that the mice fed proteins remained insulin sensitive, whereas insulin sensitivity tended to be reduced in the obese mice fed sucrose even though the difference between sucrose and protein fed mice did not reach statistical significance (Fig. 2B).

### Sucrose does not reduce the ability of fish oil to prevent diet-induced accumulation of fat in the liver

**Figure 3:**
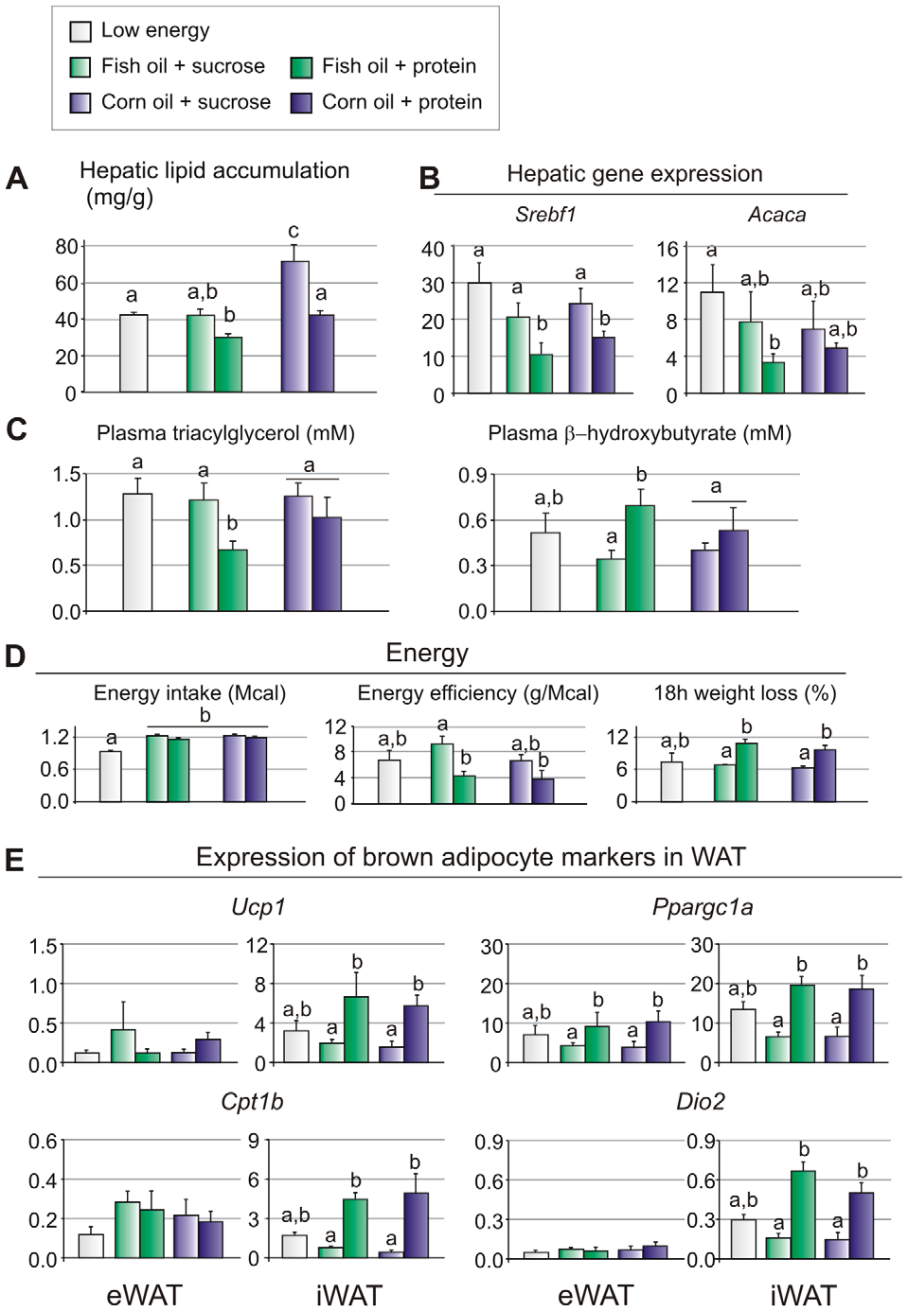
Fish oil prevents diet-induced accumulation of fat in the liver. A: Total lipids were extracted from liver and separated using HPTLC. B: Expressions of lipogenic genes (*Srebf1* (sterol regulatory element binding transcription factor 1) and *Acaca* (acetyl-Coenzyme A carboxylase alpha) were measured by RT-qPCR. C: Plasma triacylglycerol and β-hydroxybutyrate were measured in the fed state. D: Energy efficiency was calculated based on energy intake and body weight gain. E: Expression levels of brown adipose tissue marker genes (*Ucp1* (Uncoupling protein-1), *Ppargc1a* (peroxisome proliferator-activated receptor gamma, coactivator 1 alpha), *Cpt1b* (carnitine palmitoyltransferase-1b) and *Dio2* (deiodinase, iodothyronine, type II) were measured in white adipose tissues using RT-qPCR. Data are presented as means ± SEM (n = 8). Different small letters denote significant differences between the groups, in 3E within the same tissue (P<0.05).

As the anti-inflammatory effect of n-3 PUFAs in adipose tissue is well documented, we investigated if the high level of dietary sucrose reduced uptake of n-3 PUFAs. Thus, GC-MS analyses were performed to determine the fatty acid composition in red blood cells, liver and adipose tissues. These analyses demonstrated the expected enrichment of n-3 PUFA in lipids in red blood cells and liver (Table 3 and 4). In adipose tissue, the enrichment of n-3 was actually higher in mice fed the sucrose-based fish oil diet than in the protein-based fish oil diet group (Table 4). However, inclusion of sucrose in the diet did not reduce the ability of fish oil to prevent accumulation of fat in the liver (Fig. 3A). When sucrose was included in the diet, lipid accumulation in livers from fish oil fed mice was significantly lower than in livers from mice fed corn oil (Fig. 3A). Moreover, expressions of lipogenic genes seem to be determined by the sucrose∶ protein ratio independent of fat source (Fig. 3B). Thus, the ability of fish oil, but not corn oil, to protect against diet-induced lipid accumulation in the liver did not seem to be directly related to the suppression of lipogenic gene expression.

**Table 3:** Fatty acid composition in red blood cells.

		High fish oil		High corn oil	
	Low energy	Sucrose	Protein	Sucrose	Protein
**Sum total** (mg/g)	**3.41±0.04**	**3.04±0.07**	**2.87±0.05**	**3.52±0.11**	**3.34±0.10**
**SFA** (mg/g)	**1.28±0.02**	**1.24±0.03**	**1.17±0.02**	**1.31±0.05**	**1.21±0.04**
**MUFA** (mg/g)	**0.49±0.01**	**0.34±0.01**	**0.35±0.01**	**0.41±0.01**	**0.40±0.01**
**PUFA** (mg/g)	**1.39±0.02**	**1.28±0.03**	**1.19±0.02**	**1.53±0.05**	**1.44±0.04**
n-3 (mg/g)	0.25±0.00	0.79±0.02	0.73±0.01	0.21±0.01	0.11±0.00
n-6 (mg/g)	1.14±0.02	0.49±0.01	0.47±0.01	1.32±0.05	1.34±0.04
n-3/n-6	0.22±0.00	1.62±0.01	1.55±0.28	0.16±0.00	0.08±0.00

Data are presented as mean ± SEM (n = 8).

**Table 4:** Fatty acid composition in organs.

		High fish oil		High corn oil	
	Low energy	Sucrose	Protein	Sucrose	Protein
**Liver**					
**Sum total (mg/g)**	**45±4**	**41±1**	**35±2**	**71±10**	**42±3**
*%*	*100*	*100*	*100*	*100*	*100*
**SFA (mg/g)**	**15±1**	**15±0**	**12±1**	**21±3**	**13±1**
*%*	*32.5±0.9*	*36.5±0.4*	*35.2±0.3*	*29.6±0.3*	*31.3±0.8*
**MUFA (mg/g)**	**13±2**	**6±1**	**4±0**	**19±4**	**7±1**
*%*	*29.1±1.6*	*14.9±2.3*	*11.9±0.7*	*25.6±1.9*	*17.2±1.3*
**PUFA (mg/g)**	**16±1**	**19±1**	**18±1**	**29±3**	**20±1**
*%*	*36.7±1.6*	*47.2±2.0*	*51.2±0.4*	*42.7±1.9*	*47.8±0.9*
n-3 (mg/g)	3.24±0.27	12.94±0.51	10.83±0.72	3.28±0.15	1.72±0.11
*%*	*7.25±0.45*	*31.41±1.25*	*31.34±0.35*	*4.97±0.53*	*4.10±0.28*
n-6 (mg/g)	13±1	6±0	7±0	26±3	18±1
*%*	*29.4±1.2*	*15.8±0.8*	*19.9±0.5*	*37.7±1.4*	*43.7±0.7*
n-3/n-6	0.25±0.01	2.00±0.03	1.58±0.05	0.13±0.01	0.09±0.01
**eWAT**					
**Sum total (mg/g)**	**871±14**	**841±17**	**761±78**	**840±27**	**747±43**
*%*	*100*	*100*	*100*	*100*	*100*
**SFA (mg/g)**	**198±6**	**250±8**	**208±23**	**155±8**	**120±7**
*%*	*22.7±0.3*	*29.7±0.5*	*27.3±0.6*	*18.4±0.6*	*16.1±0.2*
**MUFA (mg/g)**	**372±9**	**269±6**	**268±26**	**305±7**	**279±12**
*%*	*42.7±0.4*	*32.1±0.7*	*35.4±1.1*	*36.4±0.6*	*37.5±0.8*
**PUFA (mg/g)**	**295±2**	**308±8**	**271±29**	**375±14**	**341±24**
*%*	*33.9±0.6*	*36.6±0.5*	*35.5±0.7*	*44.5±0.4*	*45.4±0.7*
n-3 (mg/g)	18.16±0.47	114.89±6.64	87.07±13.22	8.68±0.32	6.25±0.54
*%*	*2.08±0.04*	*13.63±0.59*	*11.20±0.89*	*1.03±0.02*	*0.83±0.04*
n-6 (mg/g)	277±2	190±3	181±18	366±14	322±22
*%*	*31.8±0.6*	*22.6±0.5*	*23.9±0.6*	*43.5±0.4*	*44.6±0.7*
n-3/n-6	0.07±0.00	0.60±0.03	0.47±0.05	0.02±0.00	0.02±0.00

Data are presented as mean ± SEM (n = 8).

Other hallmarks of n-3 PUFA actions are their ability to increase fatty acid oxidation and to reduce plasma triacylglycerol levels [27], [28]. Plasma triacylglycerol levels were significantly reduced in mice fed fish oil in combination with proteins, but inclusion of sucrose abrogated this effect (Fig. 3C). The higher plasma levels of β-hydroxybutyrate in mice fed fish oil in combination with proteins indicated that hepatic fatty acid oxidation was increased in these mice, and inclusion of sucrose attenuated this effect (Fig. 3C). Together these results demonstrate that the protein∶sucrose ratio also affects the ability of fish oil to reduce plasma levels of triacylglycerol and increase fatty acid oxidation.

### Increasing the protein∶sucrose ratio in a high fat diet decreases energy efficiency irrespective of corn or fish oil supplementation

To verify that the obesity in mice fed fish oil in combination with sucrose was simply not due to increased energy-intake, feed intake was recorded and energy efficiency calculated. Obviously, energy intake was significantly higher in mice fed high fat diets than that of mice receiving the low fat diet (Fig. 3D). Energy intake tended to be higher in mice receiving the sucrose diets than in mice fed the protein-based diets, but this was not statistically significant (Fig. 3D). Thus, energy efficiency was dramatically increased in mice receiving sucrose compared to protein, indicating difference in energy expenditure. A simple way to detect differences in catabolic rate is to subject mice to fasting and measure the resulting weight loss. Figure 3D shows that mice on the protein-based diets lost significantly more weight during 18 h of fasting. This supports the notion that energy expenditure is higher in mice on a protein-based diet irrespective of whether the diet is supplemented with corn oil or fish oil.

Expression and activation of UCP1 in brown and white adipose tissue lead to dissipation of energy in the form of heat, and may thus protect against diet induced obesity [29]. Gene expression analyses of adipose tissues demonstrated that expression of *Ucp1* (uncoupling protein-1), in mice fed high protein was higher in iWAT, but not in eWAT or (iBAT) (Fig. 3E). Increased expression of *Ucp1* in iWAT in mice fed the protein-based diets was accompanied by increased expression of *Cpt1b* (carnitine palmitoyltransferase-1b), *Ppargc1a* (peroxisome proliferator-activated receptor gamma coactivator 1 alpha) and *Dio2* (deiodinase, iodothyronine, type II), suggesting that iWAT adopted a more brown-like phenotype (Fig. 3E). Thus, the lean phenotype in mice fed the high protein diets, appears, at least in part, to result from increased uncoupled respiration in iWAT.

### The obesogenic effect of fish oil is determined by the macronutrient composition in *pair-fed* mice

**Figure 4:**
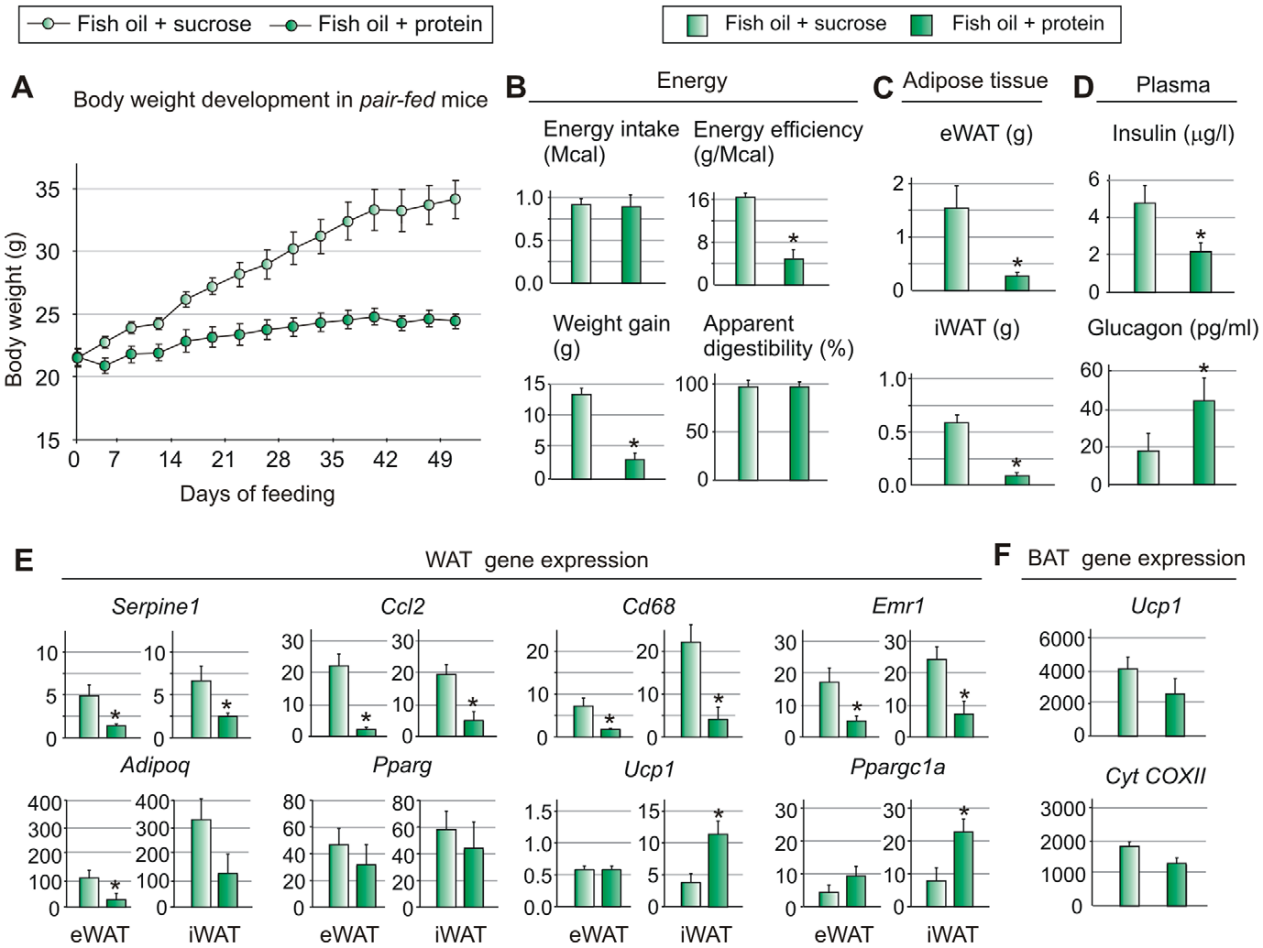
Sucrose counteracts the obesity-reducing effect of fish oil in *pair-fed* mice. Male C57BL/6 mice (n = 8) were *pair-fed* isocaloric high fish oil diets with different carbohydrate and protein contents for 8 weeks. A: Body weight development was followed throughout the feeding regime. B: Energy efficiency was calculated based on energy intake, weight gain and apparent digestibility. C: The weights of different adipose tissue depots were recorded. D: Insulin and glucagon levels were measured in plasma in the fed state. E: Inflammation and adipocyte marker genes (*Pparg* (peroxisome proliferator-activated receptor γ), *Adipoq* (adiponectin), *Serpine1* (Plasminogen activator inhibitor-1), *Ccl2* (chemokine (C-C motif) ligand 2), *Emr1* (EGF-like module containing, mucin-like, hormone receptor-like sequence 1 or F4/80) and *Cd68* (CD68 antigen) and F: thermogenesis-related genes (*Ucp1* (Uncoupling protein-1) and *cyt COXII*, (cytochrome c oxidase, subunit II) were measured by RT-qPCR in adipose tissues. Data are presented as means ± SEM. Different small letters denote significant differences between the groups, in 4E within the same tissue (P<0.05).

Since energy intake was slightly higher in mice receiving fish oil in combination with sucrose compared with protein, we decided to demonstrate directly that this difference was insufficient to account for the increased adipose tissue mass. Accordingly, mice were *pair-fed* the isocaloric diets containing fish oil in combination with sucrose or protein. To achieve identical energy intake we recorded the *ad libitum* feed intake of mice receiving the protein-based diet, and restricted the amount of feed to mice receiving the sucrose-based feed accordingly. Figure 4A demonstrates that even under conditions of *pair-feeding*, the mice receiving high sucrose gained dramatically more weight than those receiving a high protein diet. Similarly, as observed in *ad libitum* fed mice, energy efficiency and adipose tissue mass were significantly higher when mice were fed sucrose (Fig. 4B and C). Moreover, energy content in the feces was similar in both groups and the apparent digestibility was not increased by increased sucrose amount in the diet (Fig. 4B). Plasma levels of insulin were higher and glucagon lower in mice fed the sucrose than protein (Fig. 4D). In iWAT, but not eWAT, we observed a significant induction of *Ppargc1a* and *Ucp1* expression indicative of the transformation of iWAT into a more brown-like depot in protein fed mice (Fig. 4E). In iBAT, expressions of *Ucp1* and *cyt COXII*, (cytochrome c oxidase, subunit II) a marker of mitochondrial content, were not significantly different in mice fed protein or sucrose (Fig. 4F).

**Figure 5:**
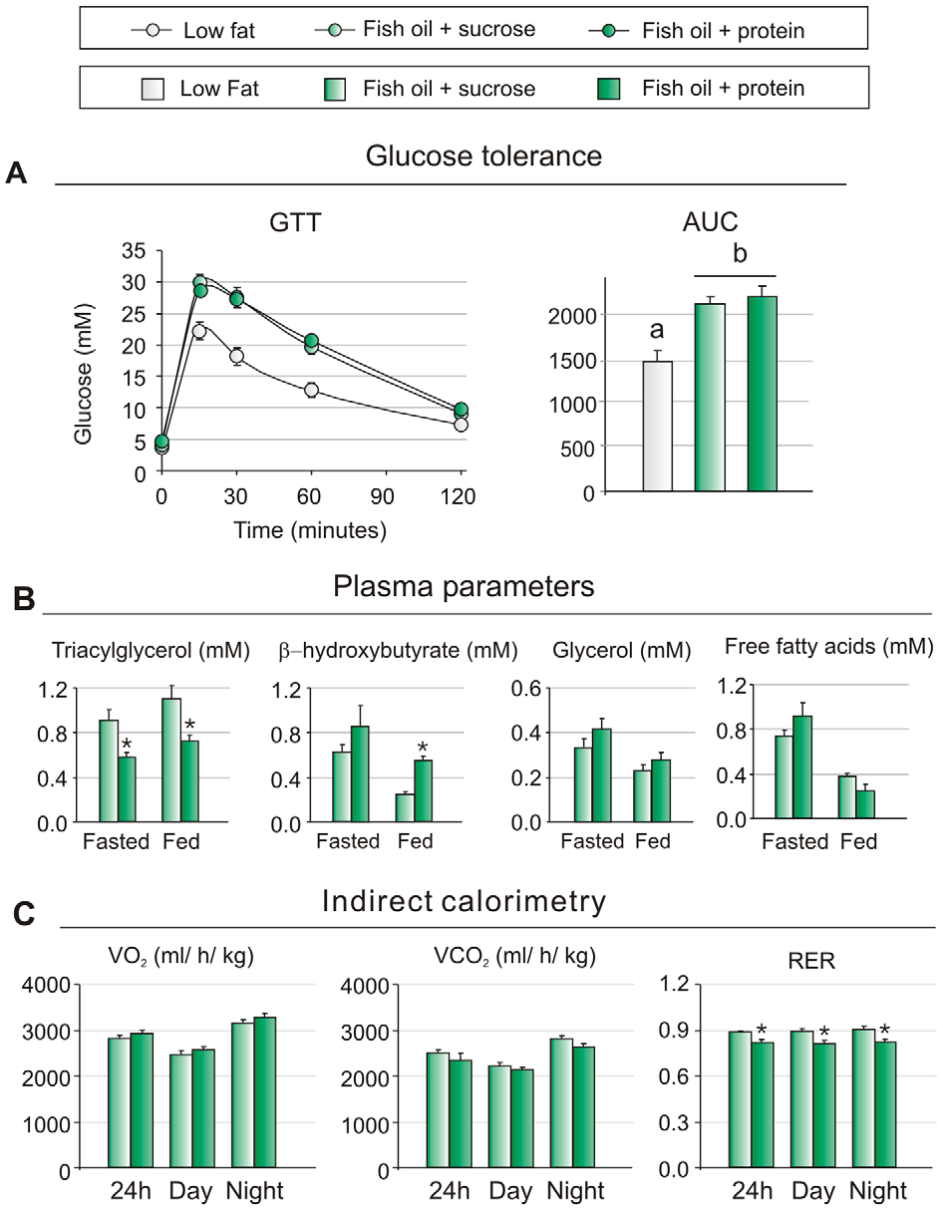
Metabolic parameters in mice fed fish oil in combination with sucrose or protein. A. Intraperitoneal glucose tolerance test was performed in mice pair fed fish oil enriched diets (n = 10). B. β-hydroxybutyrate, triacylglycerol, glycerol and free fatty acids were measured in pair-fed mice in both fasted and fed state (n = 10). C. Oxygen consumption, carbon dioxide and respiratory exchange ratio were measured during a 24-h period with indirect calorimetry (n = 8). Data are presented as means ± SEM. Different small letters denote significant differences between the groups, in 5B between fasted or fed state (P<0.05).

**Figure 6:**
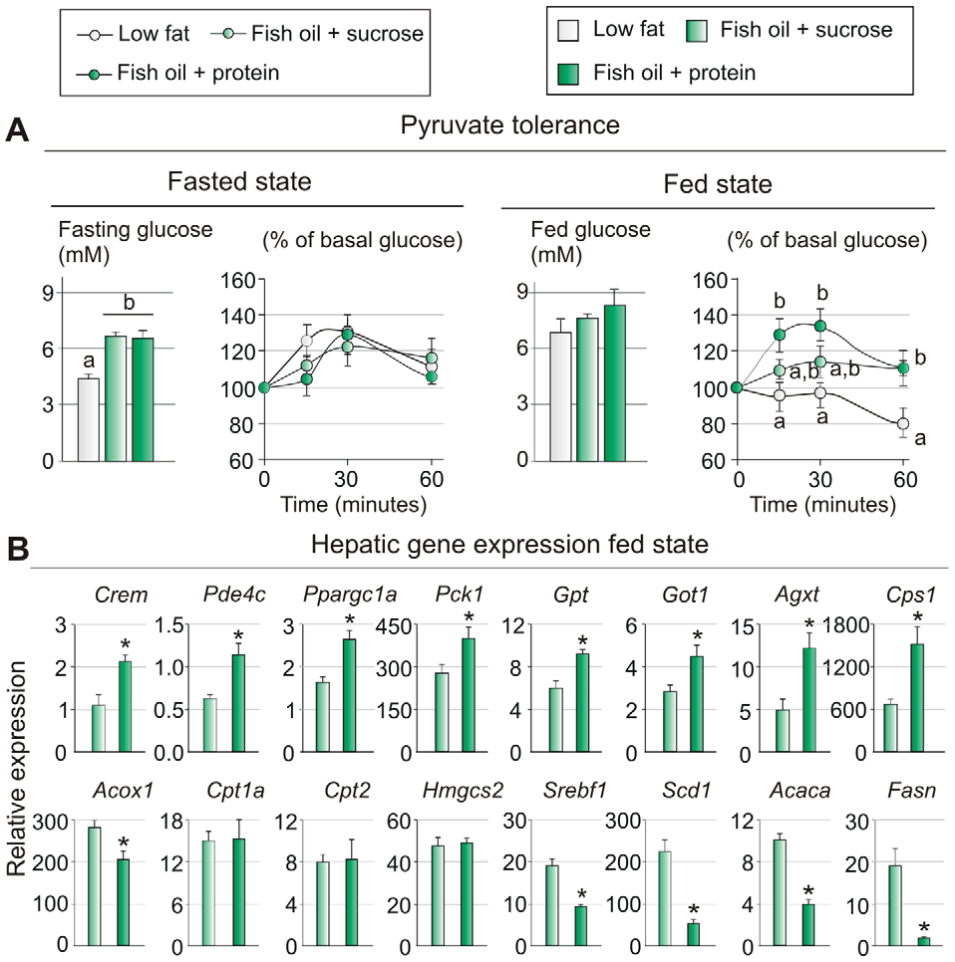
Gluconeogenesis is increased in fed state when animals are fed fish oil supplemented with protein. A. Pyruvate tolerance tests were performed on mice in 16 h fasted (n = 7) and fed states (n = 10). B. Hepatic gene expression (*Crem* (cAMP responsive element modulator), *Pde4c* (phosphodiesterase 4C, cAMP specific), *Ppargc1a* (peroxisome proliferator-activated receptor gamma, coactivator 1 alpha), *Pck1* (phosphoenolpyruvate carboxykinase 1, cytosolic), *Gpt* (glutamic pyruvic transaminase), *Got1* (glutamate oxaloacetate transaminase 1), *Agxt* (alanine-glyoxylate aminotransferase), *Cps1* (carbamoyl-phosphate synthetase 1), *Acox1* (acyl-CoA oxidase 1), *Cpt1a* (carnitine palmitoyltransferase 1a), *Cpt2* (carnitine palmitoyltransferase 2), *Hmgcs2* (3-hydroxy-3-methylglutaryl-Coenzyme A synthase 2), *Srebf1* (sterol regulatory element binding transcription factor 1), *scd1* (stearoyl-Coenzyme A desaturase 1), *Acaca* (acetyl-Coenzyme A carboxylase alpha), *Fasn* (fatty acid synthase), was measured using RT-qPCR (n = 8). Data are presented as means ± SEM. Different small letters denote significant differences between the different groups (P<0.05).

Lower expression levels of inflammatory markers in eWAT and iWAT in protein fed mice were also confirmed (Fig. 4E). In addition, as observed in *ad libitum* fed mice, glucose tolerance was similarly affected in protein and sucrose fed mice (Fig. 5A). Fasting levels of insulin were higher in sucrose+fish oil fed mice than the two other groups, but an ITT test showed no significant difference between the groups (Supp Fig. 1). Plasma levels of triacylglycerol were lower and β-hydroxybutyrate were higher in plasma from mice fed fish oil in combination with protein than in mice fed fish oil in combination with sucrose (Fig. 5B). However, expression of genes involved in fatty acid oxidation was not increased in liver (Fig. 6B) or in muscle (not shown). Actually, expression of the classical PPARα target *Acox1* (acyl-CoA oxidase 1) was higher in liver of sucrose fed mice (Fig. 6B). Thus, a possible increase in fatty acid oxidation in protein fed mice as indicated by the elevated levels of β-hydroxybutyrate did not appear to be due to increased expression of genes involved in fatty acid oxidation. Of note, however, higher expressions of *Srebf1* (sterol regulatory element binding transcription factor 1) as well as *Acaca* (acetyl-Coenzyme A carboxylase alpha) and *Fasn* (fatty acid synthase), indicate that sucrose overrides the suppressive effect of fish oil on lipogenic gene expression.

### Energy expenditure is reduced in mice fed fish oil in combination with sucrose

Mice fed fish oil in combination with sucrose exhibited increased weight gain and an increased feed efficiency indicative of decreased energy expenditure. Moreover, mice fed fish oil in combination with sucrose lost significantly less weight during 18 h of fasting (Fig. 3C). Therefore, we examined whether energy expenditure was reduced when fish oil was combined with sucrose. Accordingly, O_2_ consumption and CO_2_ production were measured by indirect calorimetry. Figure 5C shows that O_2_ consumption both in the light and the dark periods tended to be lower in mice fed fish oil in combination with sucrose than with protein. As expected, mice fed fish oil in combination with sucrose had a higher CO_2_ production resulting in a statistically significant higher RER of about 0.9 indicating a lower rate of fatty acid oxidation (Fig. 5C).

### A diet enriched with fish oil and proteins increases gluconeogenesis

High circulating levels of insulin combined with a low level of glucagon translate into reduced cAMP signalling in the liver. Thus, the observed reduced expressions of *Crem* (cAMP responsive element modulator), *Pde4c* (phosphodiesterase 4C, cAMP specific), *Ppargc1a* and *Pck1* (phosphoenolpyruvate carboxykinase 1, cytosolic) as well as reduced expressions of enzymes involved in amino acid degradation in the liver of sucrose fed mice were anticipated (Fig. 4D). In the liver PGC1α is induced in response to elevated levels of cAMP and plays a central role in the control of hepatic gluconeogenesis [30]–[32]. In keeping with the increased expressions of *Ppargc1a* and *Pck1* in liver from mice fed fish oil in combination with protein compared to sucrose, we anticipated that gluconeogenesis was induced in the fed state in the protein fed mice. To measure gluconeogenesis *in vivo* mice fed fish oil in combination with either protein or sucrose were intraperitoneally injected with pyruvate both after overnight fasting and in the fed state, and blood glucose was measured in the following 60 minutes. In the fasted state, mice fed sucrose or protein exhibited similar excursions, indicating similar rates of gluconeogenesis (Fig. 6A). In fed mice, however, the rise of blood glucose following the injection of pyruvate was dramatically faster and reached much higher levels after 15 and 30 min in the protein fed mice than in chow fed mice (Fig. 6A). Compared with chow fed mice the rise in blood glucose was also increased in mice fed fish oil in combination with sucrose, but this was not statistically significant (Fig. 6A). The decline in blood glucose in chow fed mice remains to be explained, but this was observed consistently. Taken together these results strongly support the assumption that gluconeogenesis is markedly induced in mice fed the protein-based diet.

## Discussion

It is well documented that inclusion of n-3 PUFAs in high fat diets leads to reduced development of diet-induced obesity in rodents [7]–[9], [11]–[13], [33]. Unfortunately, not all studies where the anti-obesogenic effects of fish oils are studied provide a detailed description of the macronutrient composition. However, in standard commercial available high fat- and very high fat diets, starch is the most abounded carbohydrate source and the amount of sucrose is low or absent. Here we show that a high amount of sucrose in the diet counteracts the obesity-reducing effect of fish oil as well as the well described anti-inflammatory effect in adipose tissue [19], [23], [34], [35]. Irrespective of the fatty acid source, mice fed high protein diets remained lean whereas mice fed diets enriched in sucrose became obese and had higher expressions of inflammatory markers in adipose tissue. Collectively, our results demonstrate that a high intake of sucrose abrogates the protective effects of fish oil in development of obesity.

As dietary sucrose, but not protein or fat, stimulates secretion of insulin from pancreatic β-cells, an increased dietary sucrose∶protein ratio will translate into an increased insulin∶glucagon ratio in the fed state. In this respect the observed higher insulin∶glucagon ratio in mice fed the sucrose-based diets than in mice fed the protein-based diets was expected. Increased levels of insulin in fed mice were observed irrespectively of the type of fat in the diet. Insulin is a powerful anabolic hormone that stimulates adipocyte differentiation and adipose tissue expansion [36]. Activation of insulin signaling is crucial for the development of obesity [37] and insulin receptor substrate-1 (IRS-1) transgenic mice are obese [38]. Increased insulin signaling and glucose uptake in adipose tissue in the fed state in sucrose fed mice may thus override the protective effect of fish oil when it comes to protection against obesity-development. It should also be mentioned that although several studies have demonstrated a protective effect of fish oil in obesity-development, it has been reported that inclusion of fish oil increased the amount of adipose tissue mass in hyperinsulinemic *ob/ob* mice [19].

Differences in the insulin∶glucagon ratio and hence differences in cAMP-dependent signaling may at least in part orchestrate the observed differences in energy homeostasis between the sucrose- and protein-based diets regardless of whether these diet are supplemented with corn oil or fish oil. In the liver, *Ppargc1a* is induced in response to elevated levels of cAMP and plays a central role in the control of hepatic gluconeogenesis [30]–[32]. High circulating levels of insulin combined with a low level of glucagon translate into reduced cAMP signalling in the liver. Thus, the observed increased gluconeogenesis in the fed state in protein fed mice may result from cAMP-mediated stimulation of *Ppargc1a* and *Pck-1* expression. Increased gluconeogenesis in the fed state may contribute to the observed lower energy efficiency in protein fed mice, as 6 ATP molecules are consumed per molecule of glucose synthesized from pyruvate, rendering gluconeogenesis an energy-consuming process. Moreover, concomitant increased expressions of *Gpt*, *Got1*, *Agxt* and *Cps1* suggest that energy consuming processes such as amino acid degradation and ureagenesis are higher in protein than sucrose fed mice. As mammals have no direct storage capacity for protein it needs to be metabolically processed immediately. The high cost of urea production and gluconeogenesis is actually often cited reasons for the higher thermic effect of protein than other macronutrients [39], [40] and this may partly explain why diets higher in protein exert a larger effect on energy expenditure than diets lower in protein [24].

A second mechanism by which a low sucrose∶protein ratio in the diet leads to reduced energy efficiency may be related to the observed expression of *Ucp1* in iWAT. Increased cAMP-signaling is known to induce adaptive thermogenesis by induction of *Ppargc1a* and *Ucp1* expression and it is well known that the UCP1 protein allows dissipation of energy in the form of heat [41]. Of note, acute or chronic upregulation of fatty acid oxidation alone, that is increased fatty acid oxidation without a concomitant uncoupling of mitochondria, has no net effect on whole-body energy expenditure or adiposity [42]. Although *Ucp1* expression was unchanged in iBAT, whole body energy homeostasis may be influenced by increased expression in iWAT. In fact, increased occurrence of brown-like adipocytes within WAT depots is a feature of mouse strains resistant to dietary obesity, such as the A/J strain [43] and reduced adiposity associated with aP2-transgenic expression of *Ucp1* is linked to increased energy dissipation in white, but not interscapular brown, adipose tissue [29]. Conversely, inhibition of diet-induced expression of *Ucp1* in iWAT in Sv129 mice by administration of a general cyclooxygenase inhibitor accentuates obesity-development [44].

Our finding that inclusion of sucrose abolishes the anti-obesity effect of fish oil seems to contradict a recent study from Sato et al. [45], as these authors demonstrated that inclusion of 5% the n-3 PUFA EPA (eicosapentaenoic acid) into a high fat-high sucrose diet reduced body weight gain in mice. The reason for this discrepancy is not clear, but different dietary compositions as well as doses and type of n-3 PUFAs may account for the different results obtained. The amount of n-3 PUFAs used in this study is slightly higher (6% n-3 fatty acids) than the 5% EPA used by Sato et al.. However, whereas Sato et al. used EPA, the n-3 PUFAs used in our study comprise a mixture (thereof 32±3 g/kg and 18±3 g/kg EPA and DHA (docosahexaenoic acid), respectively). Moreover, in fish oil as used in our study, the n-3 PUFAs are present in the form of triacylglycerols, whereas Sato et al. used purified EPA ethyl ester. It should also be mentioned that the main fat source in the diets used in our study is corn-oil rich in n-6 fatty acids, whereas Sato et al. used anhydrate milk fat containing more than 60% saturated fat. Last, the amount of sucrose used in our study is higher than the dose used by Sato et al. It is worth noting, however, that both the study by Sato et al. and our study demonstrated that sucrose did not reduce the ability of fish oil and/or EPA to prevent diet induced accumulation of fat in the liver.

A strong association between obesity and adipose tissue inflammation exists and obesity is characterized by chronic low-grade inflammation in adipose tissues [14], [15]. In light of this it may not be surprising that expression of macrophage and inflammatory marker genes was elevated in obese mice compared to lean mice. Still, as the anti-inflammatory effect of fish oil in adipose tissue is well described [19], [23], [34], [35], it was unexpected that the expression of inflammatory markers was similar in adipose tissue from obese corn oil and the fish oil fed groups. In our study the state of obesity rather than the n-3∶n-6 PUFA ratio in both feed and adipose tissues appeared to determine the expression levels of inflammatory markers in adipose tissue.

Chronic low grade inflammation plays an important role in development of insulin resistance [46], [47]. Pioneering work by Storlien et al. [48], later confirmed by several others [13], [23], [35], [49], [50] has demonstrated that n-3 PUFAs can prevent development of diet-induced insulin resistance in rodents. The insulin sensitizing effect of n-3 PUFAs is generally accepted to be related to the anti-inflammatory effect, recently demonstrated to be mediated by the GPR120 receptor [23]. Calculation of HOMA-IR indicated that mice fed high levels of proteins were more insulin sensitive than mice fed sucrose, but no significant differences was observed in an ITT. Similar to expression levels of inflammatory markers in adipose tissue, this was irrespective of whether the diets were supplemented with corn oil or fish oil. It was therefore unexpected that the GTT demonstrated that mice fed corn oil or fish oil in combination with sucrose or protein exhibited impaired glucose tolerance irrespective of whether or not the mice remained lean. It is possible that different mechanisms underlay the impaired glucose tolerance observed in the sucrose and the protein fed mice. It is likely that impaired glucose tolerance in sucrose fed mice is related to the obese state. Of note, in fish oil and protein fed mice, glucose tolerance was impaired even if weight gain and inflammation were maintained at low levels. Further studies are required to elucidate the mechanisms underlying the impaired glucose tolerance in these mice, but the possibility that adaption to a low carbohydrate intake with concomitant high hepatic gluconeogenesis and glucose output should be considered.

Seen as a whole, our study indicate that the sucrose∶protein ratio, rather than the n-6∶n-3 PUFA ratio in the diet determines development of obesity, adipose tissue inflammation and glucose intolerance. Activation of the NF-κB system appears to represent a link between obesity, inflammation of adipose tissue and insulin resistance [51]–[53]. Insulin is able to activate NF-κB by phosporylation of IκBα in different cell systems [54], thus, high levels of circulating insulin may activate the NF-κB system also in adipose tissue. Whether increased insulin levels in sucrose fed mice translated into activation of the NF-κB system in adipose tissue in these mice will require further investigation.

Together our results demonstrate that the background diet exerts a crucial influence on the ability of n-3 PUFAs to protect against development of obesity, glucose intolerance and adipose tissue inflammation. High levels of dietary sucrose counteract the anti-inflammatory effect of fish oil in adipose tissue and promote obesity development in mice. As the intake of sucrose in Western societies is high and increasing dietary intake of n-3 PUFAs is recommended by several health authorities it would be of importance to investigate whether the background diet influences the effect of fish oil also in humans.

## Materials and Methods

### Ethics Statement

All animal experiments were approved by National Animal Health Authorities (Norwegain approval identification: 1840 and 1841). Care and handling were in accordance with local institutional recommendations and rules. Adverse events were not observed.

### Animals and diets

Male C57BL/6JBomTac mice approximately 8 weeks of age were obtained from Taconic Europe (Ejby, Denmark) and were divided into groups (n = 6–10). The mice were kept at a 12 h light/dark cycle at 28°C. After acclimatization the animals were fed *ad libitum* or *pair-fed* experimental diets obtained from Ssniff Spezialdiäten GmbH (Soest, Germany) described in Table 1 and 2 for 8–10 weeks. All diets were supplemented with 3 g/kg L-cysteine, 10 g/kg choline bitartrate, 10 g/kg Vitamin mix AIN 76 A, 45 g/kg Mineral mix AIN 93 and 0.014 g/kg t-butylhydroquinone. Mice were euthanized by cardiac puncture under anesthesia with Isoflurane (Isoba-vet, Schering-Plough, Denmark) using the Univentor 400 Anaesthesia Unit (Univentor Limited, Sweeden) in the fed state and plasma prepared from blood. Tissues were dissected out, freeze-clamped and frozen at −80°C.

### Indirect calorimetry

The metabolic rate of mice was measured by indirect calorimetry in open circuit chambers of Labmaster system (TSE Systems GmbH, Germany). The animals were acclimated in the chambers for 24 hours and measured continuously for another 24 hours.

### Glucose, insulin and pyruvate tolerance testing (GTT, ITT and PTT)

GTT: mice were fasted 6 hours before intraperitoneal injection of 2 g/kg glucose in saline. ITT: mice were fasted 4 hours before i.p. injection of 0.5 Unit/kg human recombinant insulin in saline. PTT: mice in the fed state or mice that were fasted overnight were injected i.p. with of 2 g/kg pyruvate in saline. Blood was collected from the lateral tail vein at indicated time points and measured with Bayer Contour glucometer (Bayer A/S, Denmark).

### Plasma analyses

Insulin, glucagon and glucose [25] and lipid metabolites [55] were measured in plasma as earlier described.

### Lipid analyses

Total lipids were extracted from diets, red blood cells, liver and adipose tissue samples with chloroform: methanol, 2∶1 (v/v). Lipid classes were analyzed using an automated High-Performance Thin Layer Chromatography (HPTLC) system (Camaq, Switzerland) and separated on HPTLC plates coated with silica gel 60 F [55] whereas fatty acid composition of total lipids was analyzed on a capillary gas chromatograph with flame ionization detector (Perkin Elmer, USA) [56].

### Histology

Sections of adipose tissue were fixed, dehydrated, embedded in paraffin blocks, cut into 3 µm thick section and stained with eosin and hematoxylin as previously described [57]. Sections were visually examined using an Olympus BX 51 binocular microscope (System microscope, Japan), fitted with an Olympus DP50 3.0 camera.

### RT-qPCR

Total RNA was purified from mouse tissue using Trizol (Invitrogen). Reverse transcription (RT) was performed and cDNA was analyzed in duplicates by qPCR using the ABI PRISM 7700 Sequence Detection System (Applied Biosystems) as earlier described [58]. Primers for RT-qPCR were designed using Primer Express 2.0 (Applied Biosystems) and are available on request.

### Energy in faeces and diets

Energy content was determined in a bomb calorimeter following the manufacturer's instruction (Parr Instruments, Moline, IL, USA).

### Statistics

Data represent mean ± SEM. ANOVA, post hoc pairwise comparison: Student t-test (RT-qPCR analysis) or Tukey HSD test (GTT, ITT and organ weights). Newman-Keuls test (nonparametric due to non-homogenous variances) rest of data. Data were considered statistical significant when P<0.05).

## Supporting Information

Figure S1 A high fish oil diet does not impair insulin tolerance. Male C57BL/6 mice (n = 7) were fed a low fat or isocaloric high fish oil diets with different carbohydrate and protein contents *ad libitum* for 7 weeks. A: Insulin levels were measured in the fasted state. B: Intraperitoneal insulin tolerance test was performed. Data are presented as means ± SEM. Different small letters denote significant differences between the groups.
(TIF)
