# Supplementary figures and images for: Sucrose Counteracts the Anti-Inflammatory Effect of Fish Oil in Adipose Tissue and Increases Obesity Development in Mice
Source: PLoS One. 2011 Jun 28;6(6):e21647. doi: 10.1371/journal.pone.0021647 (PMC3125273; doi:10.1371/journal.pone.0021647)

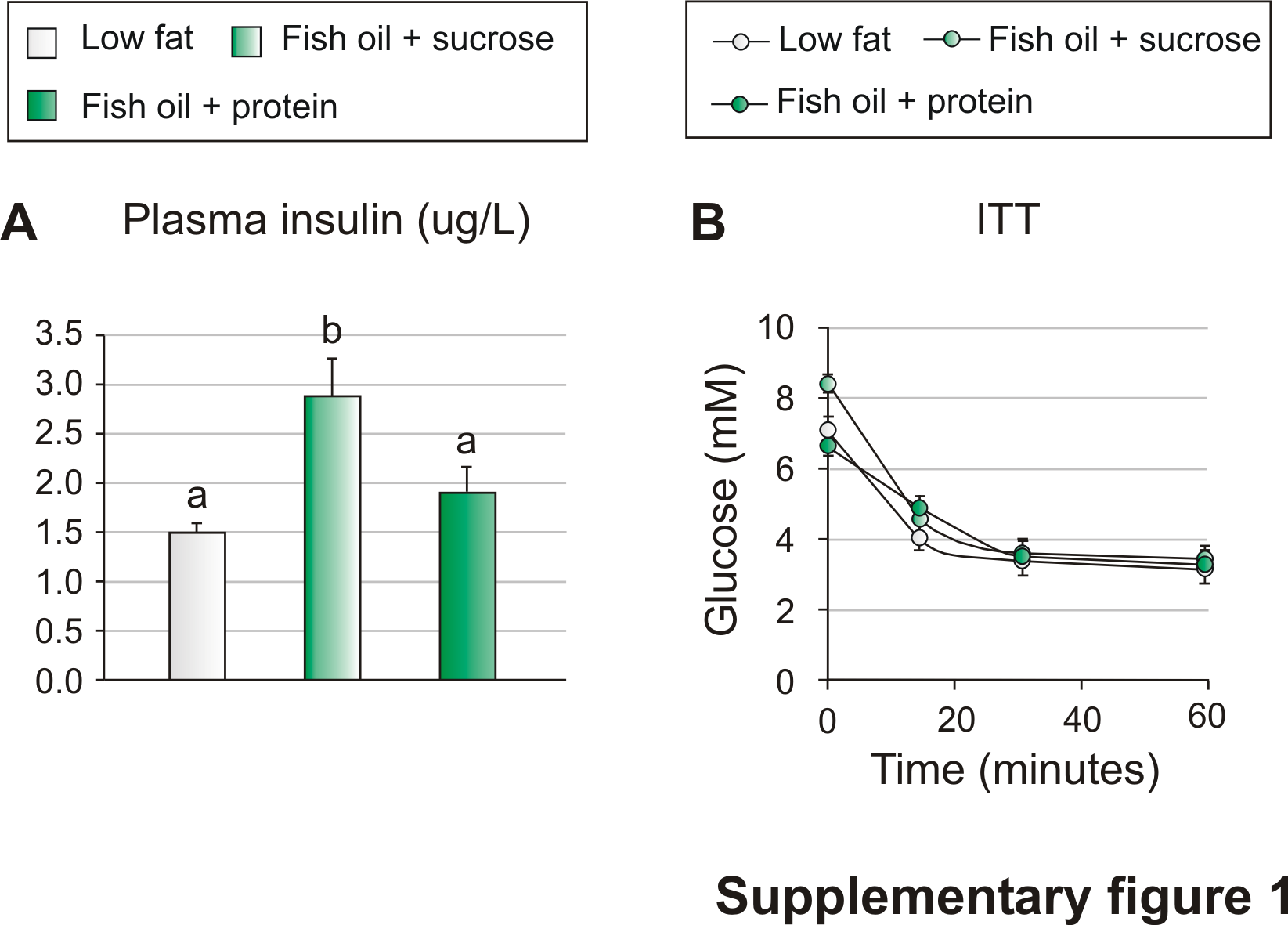

Supplement: Figure S1 — A high fish oil diet does not impair insulin tolerance. Male C57BL/6 mice (n = 7) were fed a low fat or isocaloric high fish oil diets with different carbohydrate and protein contents ad libitum for 7 weeks. A: Insulin levels were measured in the fasted state. B: Intraperitoneal insulin tolerance test was performed. Data are presented as means ± SEM. Different small letters denote significant differences between the groups. (TIF) [file pone.0021647.s001.tif]
